# Fragile Connectedness in Caregiver‐Adolescent Relationships Confers Risk for Diminished Well‐Being

**DOI:** 10.1111/famp.70131

**Published:** 2026-02-19

**Authors:** Gregory M. Fosco, John K. Coffey

**Affiliations:** ^1^ Human Development and Family Studies Pennsylvania State University State College Pennsylvania USA; ^2^ Edna Bennett Pierce Prevention Research Center Pennsylvania State University State College Pennsylvania USA; ^3^ School of Social and Behavioral Sciences Arizona State University Phoenix Arizona USA; ^4^ Yale University New Haven Connecticut USA

**Keywords:** daily diary methods, flourishing, fragile connectedness, parent–adolescent relationships, psychopathology

## Abstract

Adolescent fragile connectedness with caregivers is a process in which, rather than experiencing a consistent sense of connection with caregivers, adolescents' feelings of closeness and connection are highly reliant on the day‐to‐day experiences with caregivers. In the current study, we hypothesized that fragile connectedness would be a risk factor for internalizing psychopathology outcomes (depression, anxiety) and positive well‐being (flourishing, psychological well‐being), either as a main effect or by moderating the relation between general caregiver‐adolescent connectedness and long‐term outcomes. This study presents a secondary analysis of data from a sample of 150 9th and 10th grade adolescents (61.3% girls) in 9th and 10th grade (*M*_age_ = 14.61) living in two‐caregiver families in the Mid‐Atlantic region of the US. Adolescents and caregivers completed a baseline assessment, 21‐day daily diary burst, and 1‐year follow‐up assessment. Adolescents reported on daily connectedness with caregivers, depression, anxiety, psychological well‐being, and flourishing. Caregivers reported on daily positive parenting. Fragile connectedness was measured as individual differences in the within‐person relations between daily variation in positive parenting and adolescents' connectedness with caregivers. Outcomes were regressed on fragile connectedness, average connectedness, baseline measures of outcomes, adolescent gender, family income, living with both biological caregivers and the interaction between fragile and average connectedness. Fragile connectedness was directly associated with decreases in psychological well‐being and flourishing, but not with depression or anxiety. Average connectedness with caregivers was associated with decreases in adolescent depression over time. Implications for future research are in the emphasis on the importance of dynamic characteristics of the family (i.e., fragile connectedness) for adolescent well‐being.

## 1 Introduction

Adolescent well‐being is often narrowly conceptualized as the absence of psychopathology; however, a more complete picture emerges, one that aligns with parents primary goal for children to be happy, when we consider aspects of positive well‐being (e.g., happiness, meaning in life; Keyes 2010). A flourishing framework offers a multidimensional perspective emphasizing both high levels of positive well‐being (e.g., life satisfaction, sense of purpose) and the absence of psychopathology (Coffey et al. 2015; Keyes 2007; Suldo et al. 2016) as both important components with distinctive impact on individuals' overall mental health. Recent decades have witnessed growing adoption of this holistic view of mental health in adolescent development, in which positive well‐being and psychopathology seen as separate, yet (inversely) related constructs (Howell et al. 2016; Keyes 2007; Suldo et al. 2016). Further, research has highlighted that adolescent positive well‐being consists of multiple components such as positive emotion, life satisfaction, sense of purpose, personal growth, and social connections, similar to notions of adult well‐being (Coffey et al. 2016; Keyes 2007; Ryff 1989). In their own right, levels of positive well‐being predict future mental and physical health and success—even decades later (Boehm et al. 2023; Coffey et al. 2015). By applying a flourishing framework, we can identify risk and promotive factors that have broad implications for well‐being (e.g., factors associated with all aspects of flourishing) or specific implications for well‐being (e.g., predicting positive well‐being but not psychopathology). Thus, a flourishing framework has potential to provide a more complete and nuanced picture of adolescent developmental success and risk.

From a developmental perspective, adolescence is a period of immense psychological and physical change that can set a foundation for flourishing and/or psychopathology. During adolescence, the incidence of psychopathology increases dramatically. Data from the 2019 National Survey on Drug Use and Health indicate that 15.7% of adolescents between ages 12 and 17 reported having a Major Depressive Episode in the past year, a rate that has been climbing steadily from levels below 10% in 2011 (Substance Abuse and Mental Health Services Administration 2020). Similarly, data regarding age trends in positive well‐being indicate meaningful declines during adolescence as well (Goldbeck et al. 2007), a finding that is bolstered by short‐term longitudinal data (González‐Carrasco et al. 2017). Conversely, adolescents who are higher in well‐being are more likely to be satisfied with life, experience success, and have better health in their adult years (Boehm et al. 2023; Coffey et al. 2015, 2016). Interestingly, multi‐dimensional research examining global indicators of psychopathology and positive well‐being rarely is studied together, leaving unclear the degree to which risk and protective factors shape these distinct facets of adolescent mental health.

### 1.1 Connectedness With Caregivers: Implications for Adolescent Mental Health

Although it is common for adolescents to seek autonomy and distance from caregivers, adolescents' sense of connectedness with caregivers remains vital for their developmental success. At a fundamental level, feelings of belonging and unconditional parental regard represent a basic human need for connection (Baumeister and Leary 1995). A core component of Self‐Determination Theory (SDT; Deci and Ryan 2012), a sense of connectedness—particularly within the family—is a key factor supporting optimal development (Milyavskaya and Koestner 2011), supporting adolescents' pursuits of personally meaningful activities and interests that cultivate self‐actualization and virtue development (Deci and Ryan 2008). Other work, drawing on attachment theory, highlights how connectedness with caregivers provides a sense of security and reassurance for adolescents to feel comfortable exploring their environment and identity (Allen and Tan 2016). Adolescents who benefit from close, connected relationships with their caregivers are more likely to feel supported in their autonomy development and to continue to seek advice when navigating normative challenges with peers, academic problems, or romantic relationships, which facilitates more positive developmental outcomes (Ackard et al. 2006a, 2006b; Deci and Ryan 2012).

Adolescent connectedness with caregivers is associated with reduced risk for psychopathology and greater positive well‐being. A host of studies support this view: connectedness with caregivers is associated with lower risk for adolescents' depressive symptoms, problem behavior, substance use, and academic problems (Ackard et al. 2006a, 2006b; Fosco et al. 2012; Hochgraf et al. 2021; Preston et al. 2016; Resnick et al. 1997). Conversely, caregiver‐adolescent connectedness is also associated with elevated self‐esteem (Hochgraf et al. 2021), life satisfaction (Ma and Huebner 2008; Proctor et al. 2009), and achievement (Stewart and Suldo 2011). On a daily timescale, fluctuations in adolescents' connectedness with their caregivers corresponded to changes in their daily positive well‐being (positive affect, life satisfaction, meaning and purpose, and engagement; Fosco et al. 2020). However, most studies to date focus on static, one‐time assessments capturing average levels of connectedness with caregivers, overlooking the potential importance of changes in connectedness may have for adolescent mental health.

### 1.2 Dynamic Characteristics of the Family During Adolescence: Family Disengagement, Lability, and Fragility

The adolescent developmental period is characterized by change: contextual changes (e.g., transition into high school settings), normative strivings for autonomy, and increasing capabilities of adolescents (Steinberg and Morris 2001). Parent‐adolescent relations also change—several studies document normative declines in the degree of parental knowledge of youth activities, parental involvement, warmth, and affection with adolescents, and relationship quality (Dishion et al. 2004; Laird et al. 2003; Lippold, Fosco, et al. 2016; Lippold et al. 2018; Marceau et al. 2015, 2020). Thus, capturing the manner and degree of change in adolescent‐parent relations is an important factor in predicting long‐term outcomes.

A family disengagement perspective (Fosco and LoBraico 2019b) offers a framework for conceptualizing how individual differences in changes to adolescent‐parent relations confer risk to adolescents. More specifically, a family disengagement perspective advocates for the importance of preserving positive parenting and close relationships that are protective against risk and promotive of positive well‐being during adolescence. Although moderate declines in caregiver‐adolescent relations are normative (conceptualized as the rate of change in their linear developmental trajectory), families differ significantly in the degree to which they decline during adolescence. These individual differences in change over time have important implications for adolescent developmental outcomes. Indeed, rapid declines in parenting and family relationship quality are a key risk process for engaging in problem peer relationships, antisocial behavior, and substance use risk (Dishion et al. 2004; Laird et al. 2003; Lippold, Fosco, et al. 2016; Lippold et al. 2018; Marceau et al. 2020). More recently, similar findings are reported for internalizing problems: rapid declines in parental warmth and inclines in harsh parenting each were associated with elevated adolescent internalizing symptoms (Lippold et al. 2021). Across these studies, a key take‐away message is that *change* in family relations, over and above overall average levels of the quality of family relations, is a risk factor for poor adolescent behavioral and mental health outcomes, and sets the stage for thinking about dynamic characteristics of caregiver‐adolescent relations.

#### 1.2.1 Family Lability and Adolescent Risk

Lability is another dynamic characteristic of the family that may confer risk to adolescents. In addition to the rate of (linear) developmental change, the manner in which the caregiver‐adolescent relationship changes also impacts adolescent mental health. Specifically, lability in family relations is observed when relationships experience wide “swings” over time, observed as relatively large deviations around the linear trend over time, often operationalized as the standard deviation of a values around one's linear trend (i.e., individual Standard Deviation), or the probability of change from one measurement occasion to another across a timeseries (i.e., the mean of the squared successive differences). Adolescents in highly labile families are at elevated risk for substance use, aggression, and internalizing problems (Lippold, Fosco, et al. 2016; Lippold et al. 2018; Marceau et al. 2020; except, in some cases lability may not confer risk, see Marceau et al. 2020). Similar findings have emerged on a daily timescale: lability in daily caregiver‐child relationship quality undermines their long‐term emotional, behavioral, and physical health (Fosco et al. 2019; Lippold, Davis, Lawson, and McHale 2016). This literature converges on the idea that caregiver‐adolescent relationships are highly dynamic; and the dynamic characteristics of these relationships serve as important predictors of long‐term adolescent psychopathology outcomes. By contrast, there is a notable absence of work examining developmental lability in family relationships in relation to the positive well‐being caregivers want for their children.

#### 1.2.2 Fragile Connectedness: Implications for Adolescent Well‐Being

Recently, a new dimension of caregiver‐adolescent relationship dynamics has been described as *fragile connectedness* (Fosco and LoBraico 2019a). This term refers to a process in which adolescents' feelings of closeness and connection may be highly contingent on their daily experiences with their caregivers. Fragile connectedness is conceptualized using the same analytic framework as other studies studying affect dynamics (e.g., fragile positive affect). Daily diary studies have identified individual differences in the magnitude of the within‐person relation between daily stressors and positive affect; people who are high in fragile positive affect experience especially large decreases in positive affect on days that they experience daily stressors (Ong and Ram 2017). Fragile positive affect is a risk factor for adults' mental problems over 7 years (Rackoff and Newman 2020).

We applied the innovative methods developed around fragile positive affect to capture a within‐person contingency between daily parenting behaviors and adolescent connectedness with caregivers. The term fragile connectedness is meant to draw connections to the conceptualization of fragility found in affect dynamics, while acknowledging its distinct focus on relationship dynamics in families. We use the term *fragile connectedness* to capture the degree of change in adolescents' perceptions of closeness to caregivers in correspondence to daily variation in effective parenting behaviors (Fosco and LoBraico 2019a). Rather than experiencing a consistent sense of connectedness to caregivers, fragile connectedness is evident when adolescents experience particularly large changes in their sense of connectedness to their caregivers in relation to their response to day‐to‐day changes in caregivers' praise and encouragement.

To date, fragile connectedness has only been studied in relation to problem behaviors and substance use. In a recent study (using the same sample as the current study; Fosco and LoBraico 2019a), adolescents who exhibited higher degrees of fragile connectedness—characterized by a stronger within‐person relation between daily variation in positive parenting behaviors and feeling close and connected to caregivers—were at elevated risk for engaging in antisocial behavior, deviant peer relationships, and substance use (i.e., more frequent drunkenness, cigarette use, and marijuana use) 1 year later. However, for half of these findings, the effect of fragile connectedness was moderated by overall levels of connectedness. Specifically, fragile connectedness was a stronger risk factor for adolescents who reported generally high levels of connectedness to their caregivers. However, in the context of a generally poor relationship (low average caregiver‐adolescent connectedness), adolescents who exhibited higher levels of responsiveness to daily parenting behaviors fared better than adolescents who had a stable poor relationship with their caregivers. Thus, fragile connectedness may signal a vulnerability for disengagement in families with positive caregiver‐adolescent relationships, foreshadowing long‐term risk. Whereas for youth with generally poor relationships, the tendency to be responsive to positive daily experiences may indicate a process that is more adaptive than adolescents who have consistently weak bonds with their caregivers.

It remains unknown whether fragile connectedness also confers risk for other important domains of well‐being. When considered from a SDT lens, the fragile connectedness may reflect a risk process in which adolescents are more susceptible to experiencing thwarted belonging, which would predict more mental health problems, such as depression and anxiety (Ackard et al. 2006a, 2006b; Boutelle et al. 2009), and diminished psychological well‐being (Milyavskaya and Koestner 2011). Thus, the current study was conducted to evaluate the implications of fragile connectedness for adolescent mental health problems (i.e., depression, anxiety) and positive well‐being to address this important gap in this emerging literature. We extrapolated adolescents' responsiveness to positive parenting from a 21‐day daily diary study of 9th and 10th grade adolescents and their caregivers and conducted prospective longitudinal analyses predicting emotional distress and well‐being 1 year later. This allowed us to evaluate whether fragile caregiver‐adolescent connectedness foreshadows risk for adolescent mental health problems and declining well‐being. We hypothesized that high levels of fragility in caregiver‐adolescent connectedness would be a risk factor for increased depression and anxiety, and diminished positive well‐being. Based on prior work, we evaluated whether average levels of caregiver‐adolescent connectedness moderated the relation between adolescent fragile connectedness and long‐term mental health outcomes. We expected that, when moderation was found, fragile connectedness would be a risk factor for long‐term outcomes in the context of caregiver‐adolescent relationships that are higher in average levels of connectedness; however, fragility may not be associated with risk in the context of low caregiver‐adolescent connectedness. Rather, we expected that adolescents who had low levels of connectedness with caregivers would be at consistently elevated risk for poor mental health outcomes.

## 2 Method

### 2.1 Sample

Participants were 150 families of 9th and 10th grade adolescents who participated in part of the larger Penn State Family Life Optimizing Well‐being (FLOW) study, which was approved by the University Institutional Review Board. Families were recruited through high schools and family referrals to take part in a longitudinal study that included a daily diary assessment. Participating caregivers were those who responded to the invitation to participate in the study. This study was originally designed to capture family dynamics in two‐caregiver households (e.g., Fosco and Lydon‐Staley 2019), and relied on web‐based surveys completed nightly in homes by caregivers and adolescents requiring in‐home internet access, literacy, and English language fluency. Thus, eligibility criteria included: adolescents lived in one household continuously, adolescents lived in a two‐caregiver household, families had internet access and means to complete the daily surveys at home, participants were fluent in English, the participating adolescent was in 9th or 10th grade, and both caregiver and adolescent agreed to participate (via consent and assent). Once caregivers and adolescents consented and assented, they completed baseline surveys, a 21‐day daily diary sequence, and follow‐up surveys via web‐based, Qualtrics surveys. Data and analysis code are available upon request to the corresponding author. This study was not preregistered.

The adolescents (61.33% female) were between the ages of 13 and 16 years old (*M*_age_ = 14.61, *SD*_Age_ = 0.83). Almost all of the caregivers (95.33%) were female, and were identified as either the mother (92.67%), stepmother (1.33%), aunt (0.67%), or foster mother (0.67%) of the adolescent respondent. The remaining 4.67% of caregivers were male and identified as fathers. Caregivers were either married or lived with their partner (95.33%) or living with another caregiving adult (4.67%). Adolescent racial / ethnic background was identified (via caregiver report) as White (83.3%), Black / African American (4.7%), Asian (4.7%), Hispanic / Latino/a (0.7%), Native American / American Indian (0.7%), and Multiracial (5.3%). Most caregivers (97.32%) had at least a high school education or GED, and had completed at least some college or specialized training (82.55%). Household income ranged from “Less than $10,000” to “$125,000 or more” per year, with the median income in the sample being “Between $70,000 and $79,000” per year.

#### 2.1.1 Attrition

Of the 150 families, 9 youth did not complete the 12‐month assessment. Comparisons of demographic (e.g., sex, age, family income), baseline family factors (e.g., caregiver‐child relationship), and baseline adolescent factors (e.g., substance use, ASB) revealed only two predictors of attrition: younger caregivers (*t*(141) = −1.98, *p* = 0.05) and low child anxiety (*t*(32.40) = − 7.16, *p* < 0.001) were slightly more likely to drop out of the study. Analyses were conducted on a sample of 141 adolescents and their caregivers.

### 2.2 Measures

#### 2.2.1 Fragile Connectedness

Fragile connectedness was calculated using results from a prior study (Fosco and LoBraico 2019a), in which the within‐person relation between daily parents' positive behavior support and adolescent's feelings of connectedness with their caregivers (equation presented in Appendix S1). Data was collected from adolescents and their participating caregiver over 21 consecutive daily surveys. All items were rated on a 10‐point sliding scale (with 0.1 increments). Caregivers rated 3 items assessing *positive behavior support*. A sample item is “I praised or complimented my child for good behavior.” Adolescents rated 4 items assessing *caregiver‐adolescent connectedness* on a daily basis. A sample item is “How close and connected did you feel to your [caregiver]?” Average daily connectedness was correlated with the inventory of parent and peer attachment (*r* = 0.54, *p* < 0.01), an established measure of parent‐adolescent connectedness (Armsden and Greenberg 1987). Daily measures were evaluated to determine whether they exhibited reliable within‐person variability (*R*_*C*_; Bolger and Laurenceau, 2013) and between‐person reliability, accounting for repeated measures (*R*_*1F*_; Cranford et al. 2006). Positive behavior support exhibited reliable within‐person variability and good between‐person reliability across diary days (*R*_*1F*_ = 0.81, *R*_*C*_ = 0.58). Caregiver‐adolescent connectedness also exhibited reliable within‐person variability and good between‐person reliability across diary days (*R*_*1F*_ = 0.95, *R*_*C*_ = 0.89).

The multilevel model was estimated, and we were able to calculate individual differences in fragile connectedness as the sum of the sample average within‐person relation between positive parenting and connectedness with caregiver (i.e., fixed effects, depicted as $\gamma_{10}$ in Appendix S1) and individual differences in these values (using individuals residual scores, depicted as $u_{1i}$ in Appendix S1). The resulting score reflects strength of the relation between adolescents' connectedness with caregivers and parents' positive behavior support. Higher values reflect more fragile connectedness.

#### 2.2.2 Average Connectedness

Individuals' residual intercepts were extracted to represent the expected level of connectedness with caregivers for each adolescent (relative to the sample mean). This value is conceptually similar to an average value of connectedness across days but grand‐mean centered. Higher scores reflect higher average levels of connectedness with caregivers.

#### 2.2.3 Long‐Term Adolescent Outcomes

##### 2.2.3.1 Psychopathology

Adolescents reported on symptoms of depression and anxiety at baseline and 12‐month follow‐up assessment.

##### 2.2.3.2 Depressive Symptoms

Depressive symptoms were assessed using the 10‐item depression subscale of the Revised Child Anxiety and Depression Scale (RCADS; Ebesutani et al. 2012). Adolescents reported how often things happened to them by indicating *never* (1), *sometimes* (2), *often* (3), *always* (4) to items such as: “I feel sad or empty.” Reliability was good at baseline and follow‐up (α = 0.91, 0.92).

##### 2.2.3.3 Anxiety Symptoms

Anxiety symptoms were assessed using the 7‐item Generalized Anxiety Disorder—7 scale (GAD‐7; Spitzer et al. 2006). Adolescents rated how often in the last 2 weeks they experienced symptoms (e.g., “feeling nervous, anxious, or on edge”), from *not at all* (1), *several days* (2), *more than half the days* (3), *nearly every day* (4). Reliability was good at baseline and follow‐up (α = 0.90, 0.94).

##### 2.2.3.4 Positive Well‐Being

Adolescents reported on their positive well‐being at baseline and 12‐month follow‐up assessment.

##### 2.2.3.5 Psychological Well‐Being

Psychological well‐being was assessed using a shortened version of the Psychological Well‐Being Scale (Ryff 1989), which included 4 items from each of the 6 subscales (sample items in parentheses): self‐acceptance (“I like most aspects of my personality”), positive relations with others (“I feel like I get a lot out of my friendships”), autonomy (“My decisions are not usually influenced by what everyone else is doing”), environmental mastery (“In general, I feel I am in charge of my life”), purpose in life (“I have a sense of direction and purpose in life”), and personal growth (“I feel that I have developed a lot as a person over time”). This 24‐item shortened scale has been used elsewhere and has demonstrated to be reliable and valid (Ford et al. 2013; Neblett et al. 2008; Seaton et al. 2011). Items were rated *strongly disagree* (1), *sort of disagree* (2), *both agree and disagree* (3), *sort of agree* (4), or *strongly agree* (5). Reliability was good at baseline and follow‐up (α = 0.90, 0.92).

##### 2.2.3.6 Flourishing

Flourishing was measured using the Flourishing Scale (Diener et al. 2010), an 8‐item scale capturing adolescents' perceptions of the degree to which they experience positive mood, rewarding social relationships, optimism, engagement in life, meaning and purpose, and positive self‐evaluation (e.g., “I am a good person and live a good life”, “I am optimistic about my future”). Adolescents *strongly disagree* (1), *disagree* (2), *slightly disagree* (3), *neither agree nor disagree* (4), *slightly agree* (5), *agree* (6), *strongly agree* (7). Reliability was good at baseline and follow‐up (α = 0.93, 0.95).

## 3 Results

As a first step in our analyses, correlations, means, and standard deviations were computed for the study variables (see Table 1). Generally, correlations were in the expected directions; indices of psychopathology were positively correlated and stable over time; indices of positive well‐being also were positively correlated and stable over time; and indices of psychopathology and positive well‐being were negatively correlated. Average connectedness was negatively correlated with depressive symptoms at T2 but not significantly correlated with T2 anxiety symptoms. Average connectedness was positively correlated with T2 psychological well‐being and flourishing. Fragile connectedness was only correlated with one of the four T2 outcomes (flourishing). However, because study hypotheses were focused on whether fragile connectedness predicted *changes in* (vs. levels of) dependent variables, we also examined correlations with difference scores (T2 levels—T1 levels). In these correlations, the pattern of results changed: average connectedness was not significantly correlated with change in any of the four outcomes, but fragile connectedness was significantly associated with increases in anxiety (*r* = 0.19, *p* < 0.05) and decreases in psychological well‐being (*r* = −0.18, *p* < 0.05) and flourishing (*r* = −0.17, *p* < 0.05); the correlation between fragile connectedness and changes in depressive symptoms was not statistically significant (*r* = 0.01, *p* = 0.96). Thus, we proceeded with the analyses.

**TABLE 1:** Correlations, means, and standard deviations.

		1	2	3	4	5	6	7	8	9	10	11	12	13
1	Gender	—												
2	2‐Bio	0.07	—											
3	Fam Inc	0.06	0.25**	—										
4	Avg. connect	0.00	−0.02	−0.02	—									
5	Fragile connect	−0.05	−0.10	−0.09	−0.09	—								
6	T1 depression	−0.11	−0.01	−0.01	−0.22**	0.11	—							
7	T1 anxiety	−0.22**	−0.03	0.05	−0.09	−0.07	0.67**	—						
8	T1 PWB	0.10	−0.02	0.01	0.29**	−0.10	−0.59**	−0.49**	—					
9	T1 flourish	0.02	0.07	0.03	0.41**	−0.01	−0.54**	−0.46**	0.72**	—				
10	T2 depression	−0.14	0.01	0.04	−0.28**	0.10	0.51**	0.35**	−0.32**	−0.29**	—			
11	T2 anxiety	−0.17*	−0.06	0.13	−0.08	0.14	0.35**	0.40**	−0.27**	−0.19*	0.69**	—		
12	T2 PWB	0.14	−0.06	0.06	0.30**	−0.21*	−0.37**	−0.27**	0.60**	0.52**	−0.64**	−0.53**	—	
13	T2 flourish	−0.02	−0.11	0.09	0.25**	−0.15	−0.26**	−0.20*	0.48**	0.52**	−0.57**	−0.49**	0.86**	—
	*N*	150	149	150	150	150	150	150	150	150	140	140	140	140
	*M*	1.39	0.77	9.08	0.00	0.11	1.53	1.62	3.8	5.94	1.54	1.58	3.81	5.91
	SD	0.49	0.42	4.37	1.10	0.12	0.58	0.75	0.6	0.96	0.63	0.77	0.66	0.99

*Note:* Gender (dichotomized, higher values reflect males).

Abbreviations: 2‐Bio = two‐biological parent family status, Avg. Connect = average connectedness, Fam Inc. = family income, Fragile Connect. = fragile connectedness, PWB = Psychological Well‐Being Scale, T1 = Time 1 (baseline assessment), T2 = Time 2 (12‐month follow‐up).

* *p* < 0.05.

** *p* < 0.01.

To evaluate study hypotheses, we then computed four hierarchical linear regression models that included two steps. In the first step, we regressed T2 outcomes on T1 levels of the outcome variable, T1 levels of other outcome variables (e.g., including T1 anxiety, psychological well‐being, and flourishing in a model predicting T2 depression), fragile connectedness, and average connectedness. We included adolescent sex, family income, and living with two biological caregivers as covariates. In the second step, we added the interaction term (fragile connectedness × intercept).

### 3.1 Predicting Psychopathology Outcomes

We first computed models predicting adolescent psychopathology outcomes (presented in Table 2). The first model predicted T2 depressive symptoms. Average connectedness was associated with decreases in depressive symptoms from T1 to T2; however, this effect was qualified by the statistically significant fragile connectedness × average connectedness interaction term. In probing the interaction by generating scatterplots of the effect, the interaction appeared to be affected by two outlier cases. We recomputed the model without the outliers, and the interaction term was no longer statistically significant. Subsequent analyses were computed with and without the outliers, with no substantive effect on the results; thus, we present analyses on the full sample in the remaining analyses.

**TABLE 2:** Hierarchical regressions predicting adolescent psychopathology.

Variable	Depression		Anxiety	
	B (SE)	β	B (SE)	β
**Step 1. Direct effects**				
Baseline depression	0.492 (0.119)	0.460**	0.205 (0.150)	0.158
Baseline anxiety	0.020 (0.088)	0.024	0.280 (0.111)	0.281*
Baseline PWB	−0.009 (0.120)	−0.008	−0.153 (0.152)	0.318
Baseline flourishing	0.033 (0.075)	0.051	0.104 (0.095)	0.133
Adolescent gender	−0.100 (0.100)	−0.078	−0.149 (0.126)	−0.094
Family income	0.006 (0.011)	0.042	0.025 (0.014)	0.145
Two bio parents	0.006 (0.122)	0.004	−0.126 (0.154)	−0.066
Fragile connectedness	0.022 (0.049)	0.035	0.091 (0.062)	0.119
Average connectedness	−0.117 (0.050)	−0.192*	−0.020 (0.064)	−0.027
**Step 2. Interaction effects**				
Fragile × Average connectedness	−0.115 (0.053)	−0.167^a^	−0.046 (0.068)	−0.054

Abbreviation: PWB = psychological well‐being.

^a^ The interaction term in the depression model was determined to be due to two extreme outliers (exceeding 4.2 SD below the mean). When removed, the term was not statistically significant.

* *p* < 0.05.

** *p* < 0.01.

We then computed a model predicting changes in adolescent anxiety symptoms. In this model, fragile connectedness and average connectedness both were not associated with increases in anxiety by T2. In the second step, the interaction term (fragile connectedness × average connectedness) was not statistically significant.

### 3.2 Predicting Positive Well‐Being Outcomes

We then computed models predicting adolescent positive well‐being outcomes (presented in Table 3). The first model predicted T2 psychological well‐being. Adolescents who exhibited higher levels of fragile connectedness exhibited declines in psychological well‐being at T2. Average connectedness was not significantly linked to psychological well‐being at T2. This finding was upheld when covariates were added to the model in step 2, and the interaction term in step 2 was not statistically significant. This suggests a main effect of fragile connectedness on adolescent psychological well‐being.

**TABLE 3:** Hierarchical regressions predicting adolescent positive well‐being.

Variable	Psychological Well‐Being		Flourishing	
	B (SE)	β	B (SE)	β
**Step 1. Direct effects**				
Baseline depression	0.012 (0.111)	0.010	0.167 (0.177)	0.100
Baseline anxiety	0.031 (0.082)	0.036	−0.010 (0.131)	−0.008
Baseline PWB	0.493 (0.112)	0.448**	0.425 (0.180)	0.259*
Baseline flourishing	0.130 (0.070)	0.193	0.381 (0.112)	0.380**
Adolescent gender	0.126 (0.093)	0.093	−0.082 (0.149)	−0.041
Family income	0.009 (0.010)	0.058	0.024 (0.016)	0.109
Two bio parents	−0.150 (0.114)	−0.092	−0.403 (0.182)	−0.166*
Fragile Connectedness	−0.112 (0.046)	−0.170*	−0.152 (0.073)	−0.155*
Average connectedness	0.050 (0.047)	0.079	0.027 (0.075)	0.028
**Step 2. Interaction effects**				
Fragile × Average connectedness	−0.008 (0.050)	−0.011	−0.010 (0.081)	−0.009

* *p* < 0.05.

** *p* < 0.01.

The next model predicted adolescent flourishing. Similar to psychological well‐being, adolescents who were higher in fragile connectedness were more likely to exhibit decreases in flourishing over time; additionally, average connectedness with caregivers was not significantly associated with flourishing at T2. The interaction term tested in step 2 was not statistically significant. Again, this suggests a main effect of fragile connectedness on adolescent flourishing.

As a post hoc test, we replaced the average connectedness measure with a baseline measure of adolescent closeness with caregivers from the Inventory of Parent and Peer Attachment (Armsden and Greenberg 1987). All regression models were re‐computed with this alternative measure of average closeness, and the pattern of results regarding fragile connectedness and the interaction term between fragile connectedness and average connectedness remained the same (see Tables S1 and S2).

## 4 Discussion

Appling novel methods to 21‐day daily diary data, this study evaluated fragile caregiver‐adolescent connectedness as a developmental risk factor for adolescent depression, anxiety, psychological well‐being, and flourishing over the course of a year. Our findings underscore adolescents' day‐to‐day fragile connectedness with caregivers as a robust risk factor for diminished psychological well‐being and flourishing. Contrary to our hypotheses, fragile connectedness was not associated with adolescent depression or anxiety. Further, although average connectedness was associated with several outcomes in bivariate correlations, when evaluated in the context of fragile connectedness, average connectedness was only associated with depression but not with anxiety, psychological well‐being, or flourishing. Thus, an average sense of connectedness with caregivers was associated with reduced risk for depression over time; but fragility found in day‐to‐day interactions between adolescents and their caregivers—rather than their average sense of connectedness—was predictive of adolescent positive well‐being. Overall, this study underscores the impact of day‐to‐day experiences in the family for adolescent development and contributes to a growing literature documenting the implications of dynamic characteristics of caregiver‐adolescent relations for adolescent development and mental health (Fosco and LoBraico 2019b).

The current study extends findings in prior work (Fosco and LoBraico 2019a) documenting fragile connectedness as a risk factor for increases in problem behavior (antisocial behavior, deviant peer affiliation, conduct problems) and substance use (drunkenness, cigarette use, cannabis use) 1 year later. The current findings revealed a consistent pattern of risk in which adolescents who are higher in fragile connectedness experienced decreases in positive well‐being, which is prognostic of a host of important developmental outcomes. Indeed, when forecasting long‐term outcomes into the adult years, positive well‐being has been found to matter as much as a lack of psychopathology for outcomes such as physical health, academic outcomes, and later mental health (e.g., Boehm et al. 2023, 2022; Coffey et al. 2016; Suldo et al. 2011).

Our findings indicate that fragile connectedness is not a risk factor for depression or anxiety. We believe this pattern of results—documenting risk for positive well‐being but no evidence for internalizing psychopathology—supports the theoretical literature highlighting the need for a dual‐process framework for understanding adolescent mental health that prioritizes examining dimensions of positive well‐being and of psychopathology (Keyes 2007; Suldo et al. 2016). If positive well‐being is not evaluated as part of a broader assessment of mental health, potential risk factors in family relationships may be overlooked. As an example, in a low‐risk sample, positive well‐being was a stronger predictor of future academic success than psychological distress in a longitudinal study (Coffey et al. 2016).

As a whole, the current findings combined with prior work (Fosco and LoBraico 2019a), highlight the developmental implications of adolescent fragile connectedness at a daily level with caregivers. Adolescents who are particularly responsive to day‐to‐day changes in caregivers' positive parenting may reflect a reliance on proximal family experiences to maintain their sense of connection to caregivers, whereas adolescents who are low in fragile connectedness appear “unflappable” to the ebb and flow of positive parenting behaviors from day to day. In the context of inconsistent parenting behavior (e.g., daily fluctuations in caregiver's use of praise and encouragement), adolescents who are high in fragile connectedness are particularly vulnerable to developmental disruptions to their positive well‐being. Children with higher psychological well‐being had better adult (age 32–45) health outcomes (Boehm et al. 2022, Boehm et al. 2023). Our findings, capturing a daily family processes in caregiver‐adolescent connectedness, add to established literature linking average levels of connectedness and adolescent mental health (Ramos et al. 2022; Venning et al. 2013; Witten et al. 2019). We postulate that fragile connectedness may reflect a destabilization process in relationships that undermines feelings of security central to positive well‐being (Allen and Tan 2016; Coffey et al. 2016; Hill et al. 2020). Beyond the caregiver‐adolescent relationship, fragility may be observed in other relationships, such as romantic relationships, and during other developmental periods.

Fragile connectedness may fit within the broader developmental literature examining family disengagement processes over different timescales. The family disengagement perspective emerged out of early work documenting declines in caregiver‐adolescent relations during early and middle adolescence. During this developmental period, caregiver‐adolescent relationship quality, parental warmth, and parental involvement and knowledge of adolescent activities decline, while harsh or hostile parenting strategies tend to increase (e.g., yelling, sarcasm, insults; Dishion et al. 2004; Laird et al. 2003; Lippold, Fosco, et al. 2016; Lippold et al. 2018). This process of decline in the quality of caregiver‐adolescent relations is a critical element of premature adolescent autonomy, where *rate of change* is a key individual difference factor forecasting developmental risk. In families where declines in caregiver‐adolescent relations are gradual, presumably proceeding a developmentally appropriate pace, adolescents are at lower risk; however, in families where there is an abrupt, steep decline in caregiver‐adolescent closeness, parental monitoring, or interaction quality adolescents are at elevated risk for engaging in deviant peer relationships and escalating problem behavior (Dishion et al. 2004; Lippold, Fosco, et al. 2016; Lippold et al. 2018). Expanding the focus to consider other outcomes, decreases in connectedness with the family is also a risk factor for adolescent internalizing psychopathology (Lippold et al. 2021) and later romantic relationship quality (Fosco et al. 2016). Together, this work documents developmental changes in caregiver‐adolescent relationships as a key risk process for long‐term mental health and developmental outcomes.

Developmental change in parenting and caregiver‐adolescent relations does not always unfold in a smooth, linear fashion. In many families, family relations may be better characterized by wide swings around the family's developmental trend over time, a process described as developmental lability. Adolescents in labile families experience significant changes to the family environment where they are largely unsupervised and/or unattended at times, followed by periods when caregivers over‐correct and become especially involved and engaged, only to shift again into a state of poor monitoring and parental involvement. Adolescents in highly labile families likely experience developmentally inappropriate parental involvement and monitoring at both extremes, ultimately undermining optimal development and mental health. Lability in parental knowledge, warmth, and hostility during early to middle adolescence is a risk factor for a host of adolescent problem behaviors and internalizing problems, accounting for the rate of change over time (Lippold, Fosco, et al. 2016; Lippold et al. 2018, 2021; Marceau et al. 2015).

The current study attends to a shorter timescale, in which day‐to‐day variability in parenting and adolescent's contingent feelings of connectedness offer a window into future well‐being and risk. There is meaningful day‐to‐day variability in a wide range of family relationships, including family‐level cohesion, parental warmth, adolescent connectedness to caregivers, and couple conflict (Chung et al. 2009; Fosco et al. 2019; Fosco and Lydon‐Staley 2020; Lippold, Davis, Lawson, and McHale 2016; Lippold, Davis, McHale, et al. 2016). The current study indicates that this variability in parenting practices may undermine adolescent's sense of connectedness to the family (perhaps even functioning in a reciprocal process); and in doing so, confers risk to long‐term outcomes.

Of particular interest in the current study was the lack of support for statistical moderation between fragility and average levels of connectedness. Irrespective of the overall levels of caregiver‐adolescent relationship quality in our sample, fragility was a risk factor for psychopathology and poor mental health. Thus, it is possible that fragile connectedness captures a within‐person process that may not yet be evident in the quality of the caregiver‐adolescent relationship overall but may instead foreshadow a potentially unhealthy disengagement from the family in the coming years. These findings also point to the importance of maintaining caregiver‐adolescent relationship quality on a daily basis. From a risk assessment perspective, fragile connectedness is a within‐person process that is not captured in traditional assessments of family relations, leaving a potential risk process overlooked in most developmental models of adolescent risk.

Beyond replication of the current findings, there are important next steps to better understand the developmental nature of fragility in connectedness to caregivers during development. Within the broader family disengagement perspective, fragility may be a response to early declines in caregiver‐adolescent relations (e.g., premature autonomy processes), or even to high levels of lability in family relations that create a sense of uncertainty. Alternatively, fragility may precede developmental changes in the family disengagement, warning about impending developmental changes in the caregiver‐adolescent relationship. Future work is needed to situate fragile connectedness within the family disengagement developmental framework at other ages.

### 4.1 Limitations

There are important limitations to consider when applying the results of this study. As previously mentioned, but perhaps most importantly, replication of the effects of adolescent fragile connectedness with a new sample is important. Only one caregiver from each family participated in the current study, limiting our ability to capture fragile connectedness in each caregiver‐adolescent relationship. Future studies, collecting daily diary data from both caregivers, are needed to address this limitation. Additionally, this sample is limited in generalizability due to sampling primarily White, low‐risk families; greater racial and socioeconomic diversity when replicating these findings is important. Additionally, this study was designed to capture family process in two‐caregiver households, leaving unclear the implications in other family structures. It is possible that fragile connectedness may be an even more important risk factor for adolescents in single‐caregiver households, adolescents who have non‐resident parents, or adolescents who live in multiple households.

## 5 Conclusion

Adolescent fragile connectedness—a process in which, rather than experiencing a consistent sense of connection with caregivers, their feelings of closeness and connection are highly reliant on the day‐to‐day experiences with caregivers—is a risk factor for a host of long‐term problem outcomes for adolescents. The current study documents how adolescents who are high in fragile connectedness with caregivers are at elevated risk for decreases in positive well‐being 1 year later; yet, fragile connectedness was not associated with depression or anxiety. Rather, average connectedness predicted reduced depression 1 year later. Taken together with past work linking fragile connectedness with antisocial behavior, conduct problems, and substance use, fragile connectedness appears to be an important new index of developmental risk for adolescents.

## Funding

This study was supported by the Karl R. and Diane Wendle Fink Early Career Professorship for the Study of Families and the Penn State Social Science Research Institute (Fosco).

## Conflicts of Interest

The authors declare no conflicts of interest.

## Supporting information


**Appendix S1:** Supporting information.
