# Supplementary material for: Fragile Connectedness in Caregiver‐Adolescent Relationships Confers Risk for Diminished Well‐Being
Source: Fam Process. 2026 Feb 19;65(1):e70131. doi: 10.1111/famp.70131 (PMC12917455; doi:10.1111/famp.70131)
Supplement: Supplementary file 1 — Appendix S1: Supporting information. [file FAMP-65-0-s001.docx]

| **Table S1**  *Hierarchical Regressions Predicting Adolescent Psychopathology* | | | | | | | | |  |
| --- | --- | --- | --- | --- | --- | --- | --- | --- | --- |
|  | Depression | | | Anxiety | | |  |  |  |
| Variable | B (SE) | $\beta$ | | B (SE) | $\beta$ | | |  |  |
| Step 1. Direct effects: |  |  | |  | |  | | | |
| Baseline Depression | .494 (.121) | .462** | .210 (.150) | | .162 | | |  |  |
| Baseline Anxiety | .014 (.091) | .017 | .271 (.113) | | .272* | | |  |  |
| Baseline PWB | .014 (.124) | .013 | -.157 (.154) | | -.123 | | |  |  |
| Baseline Flourishing | .005 (.077) | .008 | .089 (.095) | | .114 | | |  |  |
| Adolescent Gender | -.103 (.102) | -.080 | -.150 (.126) | | -.095 | | |  |  |
| Family Income | .007 (.011) | .046 | .026 (.014) | | .147 | | |  |  |
| Two Bio Parents | .029 (.124) | .018 | -.124 (.154) | | -.065 | | |  |  |
| Fragile Connectedness | .031 (.050) | .049 | .093 (.062) | | .122 | | |  |  |
| Baseline Connectedness | -.063 (.059) | -.099 | .013 (.073) | | .017 | | |  |  |
| Step 2. Interaction effects: |  |  |  | |  | | |  |  |
| Fragile Connectedness*Intercept | .037 (.049) | .059 | .091 (.061) | | .118 | | |  |  |
| *Note.* Regression models were recalculated using baseline adolescent-parent connectedness (“Baseline Connectedness”) in place of the “general connectedness” variables used in the manuscript.  * *p* < .05. ** *p* < .01. | | | | | | | | |  |

**Supplemental Material**

**Multilevel Model Used to Derive Fragile Connectedness**

At level 1 (day-level variables) the equation was constructed as:

${\left( 1 \right) Connected}_{it}= \beta_{0i}+ \beta_{1i}{Day^{'}s PBS}_{it}+ \beta_{2i}{Time}_{it} + e_{it}$

Level 2 (family-level variables) as:

$${\left( 2a \right) \beta}_{0i}= \gamma_{00}+ \gamma_{01}{Usual PBS}_{i}+ \gamma_{02}{Sex}_{i}+ u_{0i}$$

${\left( 2b \right) \beta}_{1i}= \gamma_{10}+u_{1i}$

${\left( 2c \right) \beta}_{2i}= \gamma_{20}$

This model was published in Fosco and LoBraico (2019)

| **Table S2**  *Hierarchical Regressions Predicting Adolescent Positive Well-Being* | | | | | | | | |  |
| --- | --- | --- | --- | --- | --- | --- | --- | --- | --- |
|  | Psychological Well-Being | | | Flourishing | | |  |  |  |
| Variable | B (SE) | $\beta$ | | B (SE) | $\beta$ | | |  |  |
| Step 1. Direct effects: |  |  | |  | |  | | | |
| Baseline Depression | .008 (.112) | .007 | .169 (.178) | | .102 | | |  |  |
| Baseline Anxiety | .038 (.084) | .044 | -.013 (.133) | | -.010 | | |  |  |
| Baseline PWB | .487 (.115) | .443** | .415 (.182) | | .253* | | |  |  |
| Baseline Flourishing | .147 (.071) | .219* | .381 (.113) | | .380** | | |  |  |
| Adolescent Gender | .127 (.094) | .094 | -.082 (.149) | | -.041 | | |  |  |
| Family Income | .008 (.010) | .055 | .024 (.016) | | .109 | | |  |  |
| Two Bio Parents | -.159 (.115) | -.097 | -.410 (.182) | | -.168* | | |  |  |
| Fragile Connectedness | -.116 (.046) | -.177* | -.154 (.073) | | -.157* | | |  |  |
| Baseline Connectedness | .014 (.054) | .021 | .027 (.086) | | .028 | | |  |  |
| Step 2. Interaction effects: |  |  |  | |  | | |  |  |
| Fragile Connectedness*Intercept | -.062 (.0450 | -.095 | -.124 (.072) | | -.126 | | |  |  |

*Note.* Regression models were recalculated using baseline adolescent-parent connectedness (“Baseline Connectedness”) in place of the “general connectedness” variables used in the manuscript.

* *p* < .05. ** *p* < .01.

**References**

Fosco, G. M., & LoBraico, E. J. (2019b). Elaborating on premature adolescent autonomy: Linking variation in daily family processes to developmental risk. *Development and Psychopathology*, *31*(5), 1741–1755. https://doi.org/10.1017/s0954579419001032
